# Opinions about the new law on end-of-life issues in a sample of french patients receiving palliative care

**DOI:** 10.1186/s12904-016-0174-8

**Published:** 2017-01-21

**Authors:** Augustin Boulanger, Théo Chabal, Marie Fichaux, Mireille Destandau, Jean Marc La Piana, Pascal Auquier, Karine Baumstarck, Sébastien Salas

**Affiliations:** 1 Service d’Oncologie Médicale et de soins palliatifs, APHM Timone, Marseille, France; 2 La Maison, Gardanne, France; 3 Unité d’Aide Méthodologique à la Recherche Clinique et Epidémiologique, AP-HM, Marseille, France; 4 Self-perceived Health Assessment Research Unit, Aix Marseille Université, EA3279 Marseille, France; 5 Aix Marseille Université, Marseille, France; 6 Département de médecine générale, Université d’Aix-Marseille, Marseille, France

**Keywords:** Euthanasia, Opinions, Patients, Palliative care

## Abstract

**Background:**

In February 2nd 2016, the French government enacted the Claeys-Leonetti law that forbade euthanasia and established the right to deep and continuous sedation for end-of-life patients. Moreover, the law also obliges clinicians to abide by any advance directives regarding treatment and investigation, except in cases where they are “obviously inappropriate” in a given medical situation, or in cases of emergency, in order to allow medical staff to take time to assess the patient’s situation. Artificial feeding and hydration are considered as treatment. The aim of this report is to investigate individuals receiving palliative care about their opinion about euthanasia, about advance directives, about the right to deep and continuous sedation, and the right to stopping artificial feeding and hydration.

**Methods:**

The study was an opinion survey conducted among patients treated in two different palliative care institutions: a palliative care unit at the University Hospital (Timone, Marseille, France) and a non-profit association palliative care home (“La Maison”, Gardanne, France). Face-to-face interviews were performed by two investigators. The survey included sociodemographics, clinical data, and opinions about euthanasia, deep and continuous sedation, stopping artificial feeding and hydration, and advance directives.

**Results:**

Forty patients were interviewed. The mean age was 59.8 years (standard deviation 12). Fifty three percent reported opposition to legalized euthanasia. Eighty three percent were in favour of the right to deep and continuous sedation in patients with refractory pain, 75% when it concerns a patient unable to express their wishes, and 68% when the patient decides to stop vital treatment. Fifty eight percent reported that artificial nutrition and hydration should be considered as care. Fifty eight percent of the patients interviewed would like to see doctors follow the express wishes contained in advance care directives and 53% that advance directives should be subject to a validity period.

**Conclusions:**

This work demonstrates the feasibility of discussing sensitive issues such as euthanasia, continuous and deep sedation and cessation of care with patients receiving palliative care. These preliminary results point to the need to perform a larger study in order to find determinant factors in this specific situation and to incorporate them into thinking about end-of-life laws.

**Electronic supplementary material:**

The online version of this article (doi:10.1186/s12904-016-0174-8) contains supplementary material, which is available to authorized users.

## Background

The societal issues related to the end of life and euthanasia are important and have generated substantial debate. In Europe, euthanasia legislation differs from one country to another and it is legalized in only three countries [1]. For example, it has been legal in Belgium and the Netherlands since the 90s but is still prohibited in Italy and Spain. Many factors of a historical, cultural, social and religious nature may explain these disparities. In France, the question whether euthanasia should be legalized has been topical for several years. A first law (also called ‘the Leonetti Law’, April 22nd 2005) concerning the rights of patients at the end of life allows the limitation or discontinuation of treatment and sedation for a symptom that has remained refractory until death, thereby differentiating such situations from euthanasia. However, while 96% of French people have been found to be in favour of euthanasia [2], fewer than 50% of physicians favour it [3]. Moreover, most medical students were found to be in favour of it whereas palliative care specialists were largely opposed [3]. This underlines the importance of this medical and social issue in France. As a response, the French Government set up a parliamentary commission to re-examine questions related to the accompaniment of patients at the end of life and euthanasia. The commission organized the debate and many influential thinkers from the political, medical, philosophical and religious spheres were consulted by the French Parliament [4]. This broad consultation has recently given rise to a law called the Claeys-Leonetti law (2 February 2016) (Additional file 1). This law recognizes the wishes expressed by patients and establishes their rights to the following: i) deep and continuous sedation [5, 6] consisting of sedative and analgesic treatment leading to a profound and continuous change of vigilance to death if the patient is likely to suffer pain, associated with the cessation of all life-sustaining treatments such as artificial nutrition and hydration; ii) making advance directives mandatory [5, 6] as laid down in any statement written by a fully conscious patient including the decision to continue, restrict or discontinue medical treatments binding on the doctor if ever the patient is no longer able to express this decision. However, the parliamentary commission has not sought the opinion of those directly concerned, i.e. patients in palliative care. The absence of documented data from patients is partly due to the general reticence of the medical community to examine and assess fragile patients or those suffering from cognitive, psychological and/or somatic impairment. The aim of this report is to investigate individuals receiving palliative care about their opinion about euthanasia, about advance directives, about the right to deep and continuous sedation, and the right to stopping artificial feeding and hydration.

## Methods

### Design and setting

This was an opinion survey conducted among patients treated in two different palliative care units: one at a University Hospital (Timone, Marseille, France) and the other at an institution run by a non-profit association dispensing palliative care (“La Maison”, Gardanne, France, non-profit association under the terms of the 1901 law).

### Population

The inclusion criteria were as follows: over 18; with locally advanced or metastatic cancer and receiving palliative care (defined by the French Society of Palliative and Support Care, SFAP, as active care in a global approach to persons with progressive or terminal illness [7]); hospitalized in palliative care unit or in specific palliative care beds in non-palliative care units; without altered sleepiness (Epworth scale: score >16 [8]); without anxiety and/or mood disorder (HAD scale: score < 7 [9]); agreeing to participate in the study. The exclusion criteria were as follows: sedated; unable to understand the purpose and conditions of the study; unable to communicate.

### Procedure

Eligible patients were identified by the medical staff. Two investigators conducted the face-to-face interviews. Before the study began, they received training on specific problems (ethical, medical, psychological) related to this population. These sessions were run by a psychologist from the palliative health care team. Each of the two investigators chose a specific time for the interview which took into account the tiredness/sleepiness of the individual, visits from family/friends, and health care that the patient was receiving.

Before starting the interview, the investigator presented the purpose of the study and the nature of the questions to the patient. Oral consent to use data from medical records was obtained from each participant. The patient was free to accept or reject the interview. If the patient agreed to participate, He/she was then requested to answer the questions. The interview did not last longer than 30 min.

### Data collection

Data were collected from two sources: 1) medical records: socio-demographic data (age, gender) and clinical data (present hospitalization duration, nature of initial cancer); 2) face-to-face interview (Survey available in Additional file 2): pain level during the interview (visual analogic pain scale from 0 – 10; 0 no pain, 10 maximal pain) [10]; mention of believing in God; and seven specific binary questions (yes or cixno): opinion on euthanasia (defined by “a doctor intentionally killing a person by the administration of drugs, at that person’s voluntary and competent request, to end a situation judged unbearable”) (1 question), opinion on deep and continuous sedation (defined by : “consisting of sedative and analgesic treatment leading to a profound and continuous change of vigilance to death if the patient is likely to suffer pain, associated with the cessation of all life-sustaining treatments such as artificial nutrition and hydration”) (3 questions: for a patient with refractory pain, for a patient unable to express their wishes, for a patient who decides to stop vital treatment), opinion on stopping feeding and artificial hydration (1 question), opinion on advance directives (2 questions: mandatory aspect, time limited). Patients were free to develop their opinions about the questions asked in a discussion with investigators but these qualitative data were not included in this study. At the end of the survey, the investigators asked patients if any question has disturbed them or if they had comments about the survey.

### Ethics, consent and permissions

The study conformed to the principles of the Declaration of Helsinki and French Good Clinical Practices. According to French law (Article L1121-1, Law n°2011–2012 29 December 2011 - art. 5), ethical approval was not needed. All subjects participated on a voluntary basis. Consent for participation in the study was obtained from all participants.

## Results

### Population

A total of 40 patients at the end of life were interviewed. None of them rejected the investigation and all agreed to participate. Their characteristics are shown in Table 1. The sex ratio was 0.5. The mean age was 59.8 years (range: 31–84). The main cancers represented were digestive (23%), gynaecologic, head and neck, pulmonary (15.4% each) and hematologic (10.2%). The median number of days of hospitalization was ten between admission to the unit and the interview. The median pain score during the interview was 2.5/10 (range 0–8).

**Table 1:** Characteristics of the sample

		Number	Percent
Gender			
	Male	20	50
	Female	20	50
Age			
	≤50 years	10	25
	>50 years	30	75
Believe in God			
	Yes	23	57.5
	No	16	40
Type of cancer			
	Digestive	9	22.5
	Urologic	3	7.5
	Hematologic	4	10
	Gynaecologic	6	15
	ORL	6	15
	Pulmonary	6	15
	Sarcoma	1	2.5
	Melanoma	1	2.5
	Endocrinology	3	7.5
	Neurologic	1	2.5
Duration of hospitalization			
	≤10 days	17	42.5
	>10 days	14	35
Actual pain (EVA)			
	≤3	26	65
	>3; ≤6	10	25
	>6	4	10

### Opinion on euthanasia

52.5% of patients (21/40) reported opposition to legalized euthanasia for patients receiving palliative care.

### Opinion on deep and continuous sedation

Among patients, 82.5% (33/40) were in favour of the right to deep and continuous sedation when applied to refractory pain patients and 75% (30/39) when it concerns a suffering patient who is unable to express their wishes. On the other hand, only 67.5% (27/40) were in favour of deep and continuous sedation at the request of the patient who decides to discontinue vital treatment.

### Opinion about feeding and artificial hydration

57.5% of patients (23/39) reported that artificial nutrition and hydration are to be considered as care and not as a treatment.

### Opinion on advance directives

57.5% (23/40) of patients expressed the wish that advance directives be imposed on the health care team and 52.5% (21/38) were in favour of them being subject to a validity period. All results are shown in Table 2.

**Table 2:** Results

		Number	Percent
Opinion on euthanasia			
	Favourable	19	47.5
	Unfavourable	21	52.5
Opinion on deep and continuous sedation			
- For refractory pain patients able to express their wishes	Favourable	33	82.5
	Unfavourable	7	17.5
- For refractory pain patients unable to express their wishes	Favourable	30	75
	Unfavourable	9	22.5
- When patients decide to stop vital treatment	Favourable	27	67.5
	Unfavourable	13	32.5
Opinion about feeding and artificial hydration			
	Care	23	57.5
	Treatment	16	40
Opinion on advance directives			
- Advance directives be imposed on doctor	Favourable	23	57.5
	Unfavourable	17	42.5
- Advance directives are subject to a validity period	Favourable	21	52.5
	Unfavourable	17	42.5

## Discussion

This is the first study in which patients in palliative care were interviewed directly in order to assess their opinions about end-of-life conditions and euthanasia. Its main contribution is to demonstrate the feasibility of broaching sensitive issues such as euthanasia, continuous and deep sedation and cessation of care with patients in vulnerable situations. Indeed, no patient refused to participate and all contributed actively. During or following the interview, no negative feelings were expressed. However, it should be noted that anxiety and/or mood disorder was a criterion for exclusion, yet there was probably an exclusion bias in that patients’ opinions may be influenced by their HADS score.

Euthanasia is forbidden in France so in this regard the Claeys-Leonetti law has changed nothing. Nevertheless, the polls show that a large majority of French people favour the legalization of euthanasia [2] while fewer than half of French doctors are favourable to it [3]. This study suggests that patients nearing the end of life are probably more reluctant to legalize euthanasia. Unlike healthy people, such patients are directly concerned by the issue of euthanasia owing to their medical condition, a point of view that should be taken into consideration by the legislators. Furthermore, opinions expressed on euthanasia are influenced by the polls and the legal context [11, 12]. The only other study investigating the opinion of patients receiving palliative care about euthanasia was the qualitative study of Johansen et al. [13] which included 18 patients with cancer. It concluded that patients may have a positive attitude towards euthanasia but not necessarily wish it for themselves. Fear of future pain and minimal quality of life were the main reasons given for the possible wish for euthanasia.

The Claeys-Leonetti law created the right to deep and continuous sedation [6]. This right may be exercised by the conscious person in two situations: when he is a victim of refractory pain; and when he decides to discontinue vital treatment. In fact, our patients were very favourable to deep and continuous sedation, particularly in event of refractory pain. They were also favourable but to a lesser degree about the fact that a sick person at the end of life so desires it and his prognosis is very poor. The difference between the opinions in these two situations is that in the second case, the decision is not a medical decision. In France, some patients probably prefer that their own choice is not imposed on the clinician with regard to decisions affecting sedation and the discontinuation of treatment. Deep and continuous sedation also concerns patients unable to express their wishes. The aim of sedation is to prevent the suffering possibly experienced by such patients by discontinuing treatment. The questioned patients mainly approved this scenario. Doctors and health care teams should be trained in sedation and know how to follow the recommendations [14, 15]. In France, limiting or withdrawing life-sustaining treatment is not considered as euthanasia. There is a notion of intentionality to hasten end of life in euthanasia. But in the continuous sedation, the goal is to relieve suffering. The patients interviewed favoured sedation and to discontinuing treatment during sedation but most thought that artificial nutrition and hydration constituted care and not treatment. While a treatment may be suspended when it becomes useless, disproportionate or when it has no other effect than to artificially sustain life, care, whose objective is to provide wellbeing to the patient, must be maintained until death. For most patients, it was inconceivable that a doctor should have the wherewithal to suspend this and let them “die of hunger and thirst.” There is a tenacious collective vision that links water and food to health and to life. Yet when nutrition and hydration is dispensed by injection to compensate a deficient vital function, the widespread feeling in the public is that this constitutes treatment. This is especially so since they do not necessarily provide the comfort and well-being desired and can induce gastric or intestinal distension [16, 17]. In fact, the Claeys-Leonetti law stipulates that nutrition and artificial hydration are forms of treatment [6].

57.5% of the patients interviewed would like to see doctors follow the express wishes contained in advance care directives. Indeed “living wills” are a very effective means of transmitting patients’ wishes regarding the continuation, limiting or discontinuation of treatment. Patients do not wish that doctors should be in a position to challenge them because this would contravene their right to self-determination. A significant minority, however, remained committed to the opinion expressed by the doctor, not only because he or she is a professional but also a person to be trusted. In addition, the patients expressed the wish that advance directives remain subject to valid durability. According to the terms of the Leonetti law of 2005, they remain valid for 3 years from the moment they are established in writing. The Claeys-Leonetti law removed any specific duration of validity for advance care directives. They are reviewable and revocable at any time. In fact, for some patients, the law gives more power to patients than they wish to have in general.

In general, the law created the right to self-determination in this sensitive field of the end of life [12]. However, the French parliament has maintained the ban on assisted suicide (a doctor intentionally helping a person to commit suicide by providing drugs for self-administration, at that person’s voluntary and competent request) and euthanasia (a doctor intentionally killing a person by the administration of drugs, at that person’s voluntary and competent request) for seriously ill and incurable patients.

### Limitations

One of the limitations of our study and, indeed any study of the opinion of patients, in this complex area of medicine, law and ethics, is the challenge of language. Terms such as “euthanasia” “deep and continuous sedation”, “intractable pain” are often misunderstood by clinicians, legislators and the public alike. That may have included some of the patients surveyed. Equally, language is challenged by context. Every clinical situation is unique. That can be a difficult concept for non-clinicians, including patients themselves, to appreciate. Issues surrounding end of life care are complex. That complexity shall always make difficult the precise interpretation of the responses of any group of people. Certainly, any larger survey of this kind should ensure, as we attempted to do, both clarity of language and a comprehensible explanation of terms. Due to the small sample size, generalization of our findings is reduced. Larger studies should corroborate these findings in the future. Our quantitative approach should be sustained by qualitative analyses based on content analysis of face-to-face interviews that bring pertinent and essential complementary findings.

The aim of the study was to know whether this law is in line with the wishes of terminally ill patients. It shows the feasibility of discussing these issues directly with patients concerned by the law and to collect their opinions. This initial study points to the need for a large multicentre survey including a large number of patients that would establish the factors that determine what patients think about the end of life. Future legislation should take into account the opinions of terminally ill patients.

## Conclusions

This study demonstrates the feasibility of discussing euthanasia, deep and continuous sedation and advance directives with end of life patient. It reveals that they are probably more reticent to legalize euthanasia, they approve deep and continuous sedation, they consider artificial nutrition and hydration as care and they want to see their advance directives respected.

## Additional file

Additional file 1: Annexes: translation of a part of the Public health code. (DOCX 17 kb)
Additional file 2: Survey translation. (DOCX 17 kb)
