# Supplementary material for: Opinions about the new law on end-of-life issues in a sample of french patients receiving palliative care
Source: BMC Palliat Care. 2017 Jan 21;16:7. doi: 10.1186/s12904-016-0174-8 (PMC5251238; doi:10.1186/s12904-016-0174-8)
Supplement: Additional file 1: — Annexes: translation of a part of the Public health code. (DOCX 17 kb) [file 12904_2016_174_MOESM1_ESM.docx]

Annexes: translation of a part of the Public health code

1) About the sedation

Article L1110-5-2
created by LAW 2016-87 February 2, 2016 - art. 3

At the request of the patient to avoid suffering and to not undergo irrational obstinacy, deep and continues sedation causing altered consciousness maintained until death, associated with analgesia and cessation of all treatments for life support, is implemented in the following cases:

1. When the patient with a serious and incurable disease whose prognosis is engaged in short term and has a refractory suffering;

2. When the patient with a serious and incurable disease decide to stop treatment and it engage his prognosis in the short term and is likely to cause unbearable pain.

When the patient cannot express their will and to the refusal of unreasonable obstinacy mentioned in Article L. 1110-5-1, when the doctor stops treatment for life support, he or she apply deep and continues sedation causing altered consciousness maintained until death, associated with analgesia.

Continuous and deep sedation associated with analgesia as planned in this section shall be implemented according to collegiate procedure defined by regulation that allows the care team to check beforehand that the implementing conditions set out in the preceding paragraphs are met.

2) About the question of nutrition and artificial hydration

Article L1110-5-1
Created by LAW 2016-87 February 2, 2016 - art. 2

[…] Nutrition and artificial hydration are treatments that may be stopped in accordance with the first paragraph of this article. […]

3) Regarding advance directives
Article L1111-11
Edited by LAW 2016-87 February 2, 2016 - art. 8

Any adult can write advanced directives in case he would be unable to express their will one day. These advance directives express the will of the person concerning his end of life as regards of the conditions of the continuation, limiting or discontinuation of treatment or medical act.

At any time and by any way, they are reviewable and revocable. They can be written according to a model whose content is set by decree of the State Council issued after the High Authority of Health. This model provides the status of the person as it is known or not to have a severe condition when she wrote them.

Advance directives are binding on the doctor for decision of investigation, intervention or treatment, except in cases of vital emergency for the time necessary to fully evaluate the situation and when advance directives appear manifestly inappropriate or not according to the medical situation.

The decision to refuse the application of advance directives, judged by the doctor obviously inappropriate or inconsistent with the patient's medical condition, was taken after a collegial procedure defined by regulations and is registered in the medical record. It is made known to the trusted person designated by the patient or, alternatively, the family and relatives.

[…]

The attending physician informs patients of the possibility of advance directives and writing conditions.

When a person is subject to a guardianship, under Chapter II of Title XI of Book I of the Civil Code, it can write advance directives with the authorization of the judge or the family council if was formed. The tutor can neither attend nor represent on this occasion.
