# Supplementary material for: Opinions about the new law on end-of-life issues in a sample of french patients receiving palliative care
Source: BMC Palliat Care. 2017 Jan 21;16:7. doi: 10.1186/s12904-016-0174-8 (PMC5251238; doi:10.1186/s12904-016-0174-8)
Supplement: Additional file 2: — Survey translation. (DOCX 17 kb) [file 12904_2016_174_MOESM2_ESM.docx]

Additional file: Survey translation

- Do you believe in God?
  - YES
  - NO
- What is your actual pain in a scale go from 0 to 10
  - 0-10
- Euthanasia is defined by “a doctor intentionally killing a person by the administration of drugs, at that person's voluntary and competent request, to end a situation judged unbearable”.
  Are you favourable to a law permitting euthanasia in France?
  - YES
  - NO
- Deep and continuous sedation is defined by : “consisting of sedative and analgesic treatment leading to a profound and continuous change of vigilance to death if the patient is likely to suffer pain, associated with the cessation of all life-sustaining treatments such as artificial nutrition and hydration”
  Are you favourable to deep and continuous sedation in terminally phase…?
  - For a patient with a serious and incurable disease whose prognosis is short and has a refractory suffering (suffering not cured by an appropriate treatment)?
    - YES
    - NO
  - For a patient with a serious and incurable disease that decided to stop treatment, when this decision engaged his short prognosis and is likely to cause unbearable suffering?
    - YES
    - NO
  - For a patient unable to express their wish, if the doctor stops life support treatment?
    - YES
    - NO
- Do you think feeding and artificial hydration are :
  - Care? (all things helping the well-being of the patient, including hygiene, comfort, analgesia and a global care (physic, psychological, social and spiritual) of patient)
  - Treatment? (all thinks helping to cure, relieve symptoms or prevent a disease occurrence)
- Advances directives are instructions written by an adult in case he would be unable to express his will one day, it express the will of the person concerning his end of life as regards of the conditions of the continuation, limiting or discontinuation of treatment or medical act.
  - Do you want advances directives are binding on doctors?
    - YES
    - NO
  - Do you think advances directives have to be subject to a specific duration of validity?
    - YES
    - NO
- Is there any question disturbed you? Have you something to tell about this survey?
